# Cronkhite-Canada syndrome: a rare case report and literature review

**DOI:** 10.1186/s12876-016-0436-1

**Published:** 2016-02-25

**Authors:** Ruifeng Zhao, Mely Huang, Omar Banafea, Jinfang Zhao, Ling Cheng, Kaifang Zou, Liangru Zhu

**Affiliations:** Department of Gastroenterology, Union Hospital, Tongji Medical College, Huazhong University of Science and Technology, Wuhan, 430022 P. R. China

**Keywords:** Cronkhite-Canada syndrome, Polyposis, Serrated adenoma, Ectodermal abnormalities

## Abstract

**Background:**

Cronkhite-Canada Syndrome (CCS) is a rare non-inherited disease characterized by gastrointestinal polyposis and ectodermal abnormalities, the estimated incidence is about one per million. Recognizing and curing the disorder face great challenge.

**Case presentation:**

This report refers to a Chinese 52 year old man with gastrointestinal symptoms and ectodermal abnormalities. Gastrointestinal symptoms occurred without obvious cause, followed by ectodermal abnormalities after two months. In several hospitals, endoscopy examinations found numerous polypoid lesions in various sizes spreading over the stomach and the entire colon and rectum, histopathological examinations showed inflammatory and adenomatous polyp. In our hospital, both endoscopy and the contrast-enhanced computed tomography (CT) of small intestine showed gastrointestinal polyposis. Gastric antrum and the colon biopsy samples suggested hyperplastic and inflammatory polyp respectively. Endoscopic ultrasonography (EUS) suggested gastric wall thickening. Fujinnon intelligent color enhancement (FICE) revealed that the size of gastric glands pit varied, and vessels were visible. Confocal endoscope showed increased glandular epithelium layers. Magnifying narrow-band imaging endoscopy (ME-NBI) detected that pit pattern in the mucous of the polyp were regular and type III-IV of microvessels were seen. Biochemical investigations showed anemia, hypoalbuminemia and electrolyte disturbance. IgG, IgA and C3 decreased. Anti-ribosomal phosphoprotein is weak positive. The patient was given nutritional support treatment. Gstrointestinal symptoms and hyperpigmentation improved gradually.

**Conclusion:**

The patient was ever hospitalized in four hospitals and was diagnosed with CCS after 8 months of gastrointestinal symptoms. So when encountering the patient with gastrointestinal polyposis and ectodermal abnormalities, try to take CCS into consideration. Due to its low incidence, no standard therapy regimen has been established so far. However, nutritional support treatment is of great significance.

## Background

CCS is a rare non-inherited disease characterized by gastrointestinal polyposis and ectodermal abnormalities including hyperpigmentation, alopecia and onychodystrophy [1]. The diagnosis of CCS is based on history, physical examination, endoscopic findings of gastrointestinal polyposis and histopathology [2]. The etiology is not clear so far. The standard therapy regimen has not been established. Current medical therapies include various antibiotics, anabolic steroids, dietary supplementation, ranitidine, glucocorticoid, immunomodulatory agent, salazosulfapyridine, anti-tumor necrosis factor antibody and anti- Helicobacter pylori regimen [3–7], surgery is necessary when complications occur [8]. Its incidence is low whereas mortality is high.

## Case presentation

**Fig. 1:**
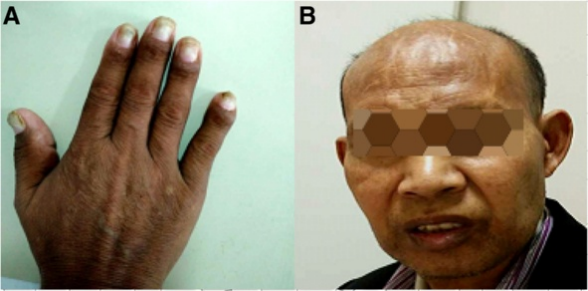
Physical findings of the CCS patient. Hyperpigmentation (**a**), onychodystrophy (**a**) and alopecia (**b**) were seen on the patient

A 52-year-old Chinese man was admitted to our hospital with 8-month history of diarrhea (4-5 loose stools actions per day, sometimes up to more than ten times), bloody stools, abdominal distension, nausea, vomiting, appetite loss and weight loss of 10 kg in the recent 8 months. Two months after the onset of gastrointestinal symptoms, skin hyperpigmentation, fragile nails and scalp hair loss occurred. In other hospitals, endoscopy examinations suggested numerous polypoid lesions in various sizes spreading over the stomach and the entire colon and rectum, repeated histopathological examinations showed inflammatory and adenomatous polyp. The patient was once considered to be lymphoma whereas was not diagnosed definitely, antibiotics and symptomatic treatments were applied, the gastrointestinal symptoms did not alleviated. The patient’s occupation was farmer and past history was unremarkable. There was no family history of gastrointestinal polyposis. Physical examination exhibited alopecia, atrophic nail changes, diffuse hyperpigmentation of the skin (Fig. 1)*.* There were no positive findings on heart, lung, abdomen and limbs.

**Fig. 2:**
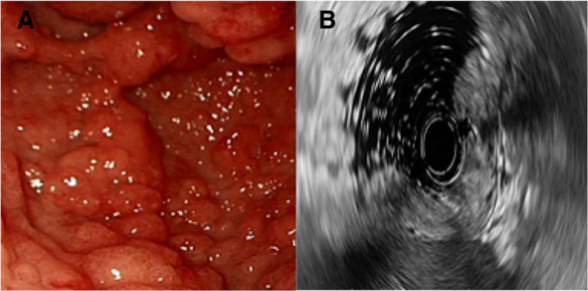
Endoscopy findings in the stomach. Multiple polyps were seen in the stomach via gastric endoscopy (**a**). EUS showed gastric wall thickening (**b**)

**Fig. 3:**
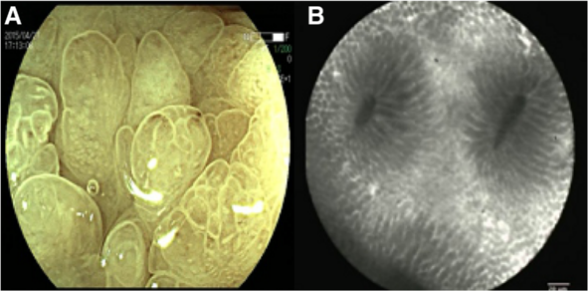
The changes of gastric glands, vessels, glandular epithelium in the stomach mucosa. FICE revealed that the size of gastric glands pit varied, and vessels were visible (**a**). Confocal endoscope showed increased glandular epithelium layers (**b**)

**Fig. 4:**
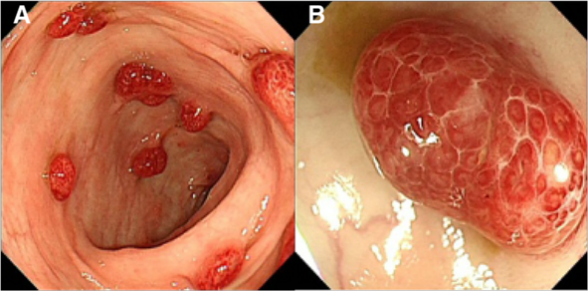
Endoscopic findings in the colon. Multiple polyps was seen in the colon via colonoscopy (**a**). ME-NBI detected the regular pit pattern of glandular tubes and type III-IV of microvessels in the mucous of the polyp (**b**)

**Fig. 5:**
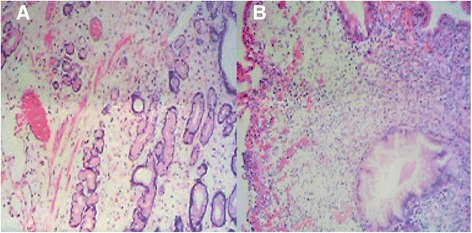
Histopathological examination of gastric and colon biopsy samples. Gastric antrum biopsy samples suggested hyperplastic polyp (**a**). Colon biopsy samples suggested inflammatory polyp (**b**)

**Fig. 6:**
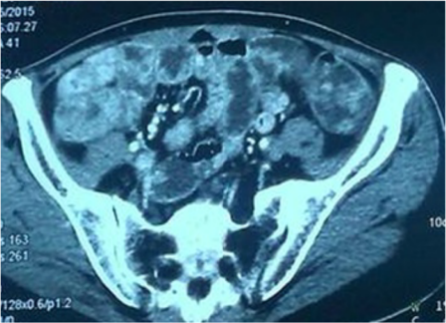
The contrast-enhanced computed tomography of small intestine. Nodular bumps spread over distalileum and entire colon

Biochemical investigations detected several abnormalities: a peripheral blood hemoglobin level of 115g/l (normal range 130-175g/l), a serum total protein level of 30.6 g/l (normal range 64–83 g/l) and a serum albumin level of 9.4g/l (normal range 20-30 g/dl). The level of serum sodium, potassium, chloride and calcium decreased. IgG, IgA and C3 decreased. Anti-ribosomal phosphoprotein is weak positive. Bacterial culture of stool found no salmonella, shigella and fungus. Cortisol secretion rhythm, CRP, ESR and IgG4 were on normal level. Upper gastrointestinal endoscopy found diffuse polyps throughout the stomach (Fig. 2a), EUS suggested gastric wall thickening (Fig. 2b). FICE revealed that the size of the gastric glands pit varied, and vessels were visible (Fig. 3a). Confocal endoscope showed increased glandular epithelium layers (Fig. 3b). Colonoscopy found diffuse polyps in the entire colon and rectum (Fig. 4a). ME-NBI detected that pit pattern of glandular tubes in the mucous of the polyp were regular and type III-IV of microvessels were seen (Fig. 4b). Gastric antrum and the colon biopsy samples suggested hyperplastic and inflammatory polyp respectively (Fig. 5). The contrast-enhanced computed tomography (CT) of small intestine displayed widespread nodular bumps over distalileum and entire colon (Fig. 6). Given the diffuse polyps in gastrointestine, the patient tended to be diagnosed with polyposis. Further, combining polyposis with ectodermal abnormalities, onset age and family history, the final diagnosis of CCS was made. The patient was given nutritional support treatment including fat emulsion and amino acid for two weeks, diarrhea relieved and hyperpigmentation improved. Then the patient discharged from the hospital, and took proton pump inhibitor orally. But the patient didn’t respond to treatment, severe diarrhea appeared again. Unfortunately the patient was died three months later.

## Discussion

CCS was first described by Cronkhite and Canada in 1955 [9]. CCS can develop in all ethnic groups, but the most frequently affected populations were European or Asian descent and most of the cases were reported from Japan [10]. The onset age was mainly between 50 and 70 years old [2]. A slight male predominance was noted [10], the ratio of male to female was about 1.3 ~ 2.3:1 [2].

The etiology of CCS remains unclear. Patients with CCS accompanied by vitiligo, systemic lupus erythematosus, scleroderma, hypothyroidism and membranous glomerulonephritis have been reported in the literature [8, 11–13], as well as CCS has been seen in association with antinuclear antibodies, which provide evidences to support CCS to be immune-mediated [11]. Histologic findings of increased autoimmune-related IgG4 antibody in CCS polyps and treatment responses to immunosuppressants are also consistent with an autoimmune mechanism underlying CCS [8]. Eric ward reported that mast cells were seen on histopathologic analysis of intestinal biopsies and a combination regimen containing H2-receptorantagonists, cromolyn sodium and loratadine was effective, which indicates the possible role of mast cell dysfunction in the pathogenesis of CCS [3]. Goto et al reported that stresses, such as excessive physical exertion and mental strain, may trigger this syndrome. He also reported a frequency (3%) of concurrent psychiatric disorders in patients with CCS [14]*.* Mental stress may cause local inflammation in the gastrointestinal mucosa [11]. CCS is characterized by gastrointestinal polyposis and ectodermal abnormalities. The polyps are characteristically distributed throughout the entire gastrointestinal tract except esophagus [1]. The histopathologic types of the polyps have recently been reported as inflammatory, non-neoplastic, or retention polyps [15]. However, gastrointestinal malignancies can be as high as 15% [1]. The neoplasms develop primarily in the colon and less frequently in the stomach [4]. Whether malignant lesions arise from preexisting polyps or coincidentally are a doubt. A study showed that serrated adenoma of the polypoid lesions was retrospectively found in 10 (40%) out of 25 CCS cases with colon cancer [15]. Serrated adenoma have potential to be malignant and is often difficult to detect by endoscopy [16]. Moreover, both serrated adenoma and hyperplastic polyps are of sessile type and possess saw-toothed, elongated, and dilated crypts. However the latter does not show significant nuclearatypia in histopathological examinations, so serrated adenoma may be considered to be hyperplastic polyps by mistake. It suggests that CCS associated with colorectal cancer may frequently have polyps containing serrated adenoma lesions [15]. Ectodermal changes may be secondary to malabsorption [11]. In the case we report, ectodermal changes occurred 2 months after the onset of gastrointestinal symptoms. With the improvement of diarrhea, hyperpigmentation relieved. However, there are a few reports in which ectodermal changes preceded the gastrointestinal manifestations.

Medical treatment regimens reported in the literature vary. Corticosteroid, anabolic steroids, various antibiotics, dietary supplementation, ranitidine, and symptomatic treatment have all been used. Patients commonly have only temporary symptomatic improvement [3]. It is reported that steroid-resistant CCS was successfully treated by cyclosporine and azathioprine [5], and a case was successfully treated by anti-tumor necrosis factor antibody after poor therapeutic effect of corticosteroids, antiplasmin agents, and azathioprine [6]. A CCS patient associated with HP infection achieved remission after treatment with anti- Helicobacter pylori *r*egimen [7]. SASP was effective in eradication of the inflammatory polyposis [4]. Surgery may be indicated for the treatment of bowel complications, such as severe protein-losing enteropathy, persistent haematochezia, malignant transformation [1, 8, 17] and rarely for removing the polyps burden [8]. The progressive course of CCS makes the mortality up to 55% [3], frequently due to complications such as anemia, gastrointestinal bleeding, congestive heart failure, and septicemia [5]. Therefore, it is important to be diagnosed early to provide appropriate treatment and follow up. The treatment outcome in case report is encouraging, but it has been not confirmed by mass of evidence-based medicine.

## Conclusion

CCS is a rare disease, physician should have a profound understanding of the characteristics of gastrointestinal polyposis including heredity factor, onset age, parenteral manifestation and histopathology to try to avoid missed diagnosis and misdiagnosis. In the aspect of treatment, nutritional support treatment is fundamental, more cases would be needed to get the optimal therapy regiment.

### The name of ethics committee and reference number

The Ethics Committee of Tongji Medical College, Huazhong University of Science and Technology; IORG No: IORG0003571.

### Ethics, consent and permission

The Ethics Committee of Tongji Medical College, Huazhong University of Science and Technology (IORG No: IORG0003571) Voted at its meeting on 20/06/2015 to give final APPROVAL for the study Cronkhite-Canada syndrome: A rare case report and literature review which is conducted by Associate Prof. Zhu liangru at Department of Gastroenterology, UnionHospital, Tongji Medical College, Huazhong University of Science and Technology.

### Consent to publish

Written informed consent was obtained from the patient for publication of this case report and any accompanying images. A copy of the written consent is available for review by the Editor-in-Chief of this journal.

## Abbreviations

CCS: Cronkhite-Canada syndrome
EUS: Endoscopic ultrasonography
CT: Computed tomography
FICE: Fujinon intelligent color enhancement
ME-NBI: Magnifying narrow-band imaging endoscopy
